# Identifying Influential Nodes in Large-Scale Directed Networks: The Role of Clustering

**DOI:** 10.1371/journal.pone.0077455

**Published:** 2013-10-31

**Authors:** Duan-Bing Chen, Hui Gao, Linyuan Lü, Tao Zhou

**Affiliations:** 1 Web Sciences Center, School of Computer Science and Engineering, University of Electronic Science and Technology of China, Chengdu, People’s Republic of China; 2 Institute of Information Economy, Alibaba Business College, Hangzhou Normal University, Hangzhou, People’s Republic of China; 3 Department of Physics, University of Fribourg, Fribourg, Switzerland; University of Maribor, Slovenia

## Abstract

Identifying influential nodes in very large-scale directed networks is a big challenge relevant to disparate applications, such as accelerating information propagation, controlling rumors and diseases, designing search engines, and understanding hierarchical organization of social and biological networks. Known methods range from node centralities, such as degree, closeness and betweenness, to diffusion-based processes, like PageRank and LeaderRank. Some of these methods already take into account the influences of a node’s neighbors but do not directly make use of the interactions among it’s neighbors. Local clustering is known to have negative impacts on the information spreading. We further show empirically that it also plays a negative role in generating local connections. Inspired by these facts, we propose a local ranking algorithm named ClusterRank, which takes into account not only the number of neighbors and the neighbors’ influences, but also the clustering coefficient. Subject to the susceptible-infected-recovered (SIR) spreading model with constant infectivity, experimental results on two directed networks, a social network extracted from delicious.com and a large-scale short-message communication network, demonstrate that the ClusterRank outperforms some benchmark algorithms such as PageRank and LeaderRank. Furthermore, ClusterRank can also be applied to undirected networks where the superiority of ClusterRank is significant compared with degree centrality and *k*-core decomposition. In addition, ClusterRank, only making use of local information, is much more efficient than global methods: It takes only 191 seconds for a network with about 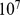 nodes, more than 15 times faster than PageRank.

## Introduction

With great theoretical and practical significance, the studies on epidemic and information spreading in biological, social and technological networks become one of the most exciting domains in many branches of sciences [1]–[4]. Therein how to control the spreading process is of particular interests [5], where the identification of influential nodes is a crucial issue according to the assumption that highly influential nodes are more likely to be infected and to infect a larger number of nodes [6]–[8].

A number of centrality indices have been proposed to address this problem, such as degree centrality, closeness centrality [9], betweenness centrality [10], and eigenvector centrality [11]. Degree centrality is a straightforward and efficient metric but less relevant. Recent researches show that top-degree ranking nodes have positive effects on cooperative behaviors in social networks [12], [13]. However, the location of a node in the network may play a more important role than its degree. For example, a node located in the center of the network, having a few highly influential neighbors, may be more influential than a node having a larger number of less influential neighbors. Considering this fact, Kitsak *et al.* [6] proposed a coarse-grained method by using 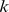-core decomposition to quantify a node’s influence based on the assumption that nodes in the same shell have similar influence and nodes in higher-level shells are likely to infect more nodes. This method may fail in some cases. For example, in a tree, all nodes are in 1-core and thus expected to have the same influence according to [6]. However, this tree may be hierarchically organized (e.g., the binary tree) and nodes near the root have much higher influence than leaves. Chen *et al.* [14] devised a semi-local index by considering the next nearest neighborhood, which can well identify influential nodes in a hierarchical tree and give more elaborate division than 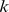-core decomposition. Experimental results demonstrated that the semi-local index performs as good as global indices while has much lower computational complexity, and thus it obtains a good trade-off on effectiveness and efficiency. Recently, Chen *et al.* [15] considered the effect of path number and path diversity while ranking the spreading ability of nodes in networks and introduced two correction factors correspondingly. The ranking accuracy is considerably increased compared with some well-known ranking methods, such as PageRank and LeaderRank.

With explosive data growth, the design of efficient and effective ranking algorithms on very large-scale networks is becoming a big challenge nowadays [16]. The representative methods include the well-known HITs [17] and PageRank [18], as well as some recently proposed algorithms like LeaderRank [7], [19] and TwitterRank [20]. All these algorithms are diffusion based (or say random-walk based), with a common assumption that a node is expected to be of high influence if it points to many highly influential neighbors (here, a link from 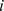 to 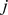 indicates that 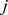 is a follower of 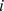). It has been demonstrated that these methods outperform out-degree centrality in terms of ranking effectiveness. In addition to the direct influential scores of neighbors, the interactions among neighbors may also play a significant role. The density of interactions among neighbors can be characterized by the local clustering coefficient [21], which has great impacts on network dynamics, such as game theory [12], [13], [22]–[24], cascading [25], synchronization [26], [27] and spreading [28]–[31]. Empirical analysis also shows that nodes with smaller clustering have higher ability to attract new connections [32], [33].

Keeping this in mind, in this paper, we propose a local ranking method, named ClusterRank, to identify influential nodes in directed networks by taking into account the effects of local clustering on information propagation. Besides the localization of our algorithm, another distinguishable difference from the above-mentioned diffusion-based algorithms is that the clustering coefficient is directly involved in the definition of a node’s influence in ClusterRank. We apply the SIR spreading model with constant infectivity to test the effectiveness of our method on four real networks, including two large-scale directed networks (a social network extracted from *delicious.com* consisting of 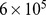 nodes and a short-message communication network containing about 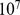 nodes) and two undirected networks (one is collaboration network from condensed matter e-print archive consisting of about 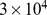 nodes [34] and the other is an undirected version of the social network from *delicious.com*). Experimental results show that ClusterRank performs much better than the simplest degree centrality, and the top-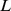 influential nodes identified by ClusterRank lead to much wider and faster spreading than those by PageRank or LeaderRank. Besides, the computations of ClusterRank on the network with 10 millions of nodes can be finished in 191 seconds by using C#.net language on a Core II 2.0 GHZ CPU processor with 2 GB memory, more than 15 times faster than PageRank algorithm.

## Materials and Methods

### 1.1 Empirical Analysis

Many social networks can be represented by directed networks where a link from 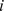 to 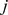 means 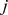 is a follower of 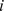, indicating that 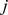 receives information from 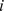. We denote 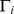 as the set of followers of 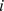 and the density of interactions among 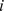
’s followers can be characterized by the local clustering coefficient of 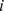. Based on the original definition of clustering coefficient [21], the clustering coefficient of node 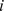 in a directed network is extended as:
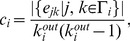
(1)where 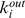 is the out-degree of 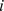, namely the number of followers of 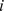, and 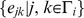 is the set of links connecting two of 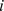
’s followers. Let 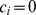 if 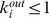. According to Eq. (1), a reciprocal link 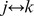 is counted as two separate links 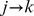 and 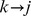.

The local clustering has remarkable impacts on network structure and functions. Studying the effects of clustering coefficient on the network evolving can provide insights into the understanding of growing mechanism and further help us to design better link prediction algorithms [35]–[37] and to explain the observation on information spreading through online social networks [30]. Some literatures showed that the clustering has negative correlation with degree in undirected networks [38] or with total degree in directed networks [39]. Here, we take two real evolving networks as examples to analyze the effect of clustering coefficient. One is a collaboration network from condensed matter e-print archive (Cond-mat for short) [34], the other is a short-message communication network (SM for short) from a mobile company in eastern China city. For each network, we consider two snapshots which contain the data starting from a given date (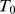) but ending with different dates (i.e., 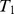 and 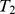 respectively). Specifically, the first network of Cond-mat is from Jan. 1st, 1995 (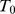) to Dec. 13th, 1999 (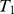) containing 16264 nodes and 47594 links, and the second network of Cond-mat is from Jan. 1, 1995 (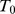) to Jun. 30, 2003 (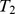) containing 30460 nodes and 120029 links. Similarly, for SM, the first network consists of the data from Dec. 8th to Dec. 17th, 2010 with 3612863 nodes and 7472808 links, and the second network is from Dec. 8th, 2010 to Jan. 6th, 2011 with 9193545 nodes and 22901318 links. Here, Cond-mat is undirected and SM is directed where a link from 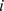 to 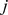 represents that 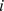 has sent at least one message to 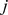.

**Figure 1:**
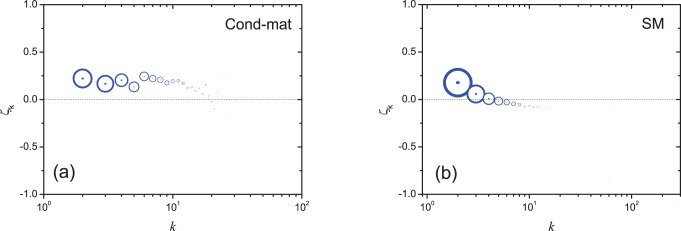
The correlation between 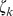 and the degree in the first network 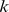. The area of a circle is proportional to the number of nodes with the corresponding degree.

**Figure 2:**
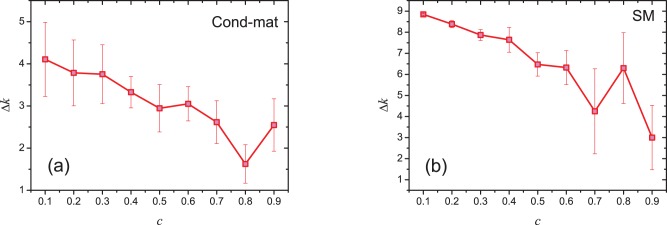
The increment of degree 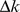 in the period 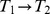 of nodes with the same degree (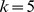) but different clustering coefficients at time 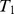. 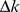
 is the average value of a bin (size = 0.1) on clustering coefficient. For example, the value of 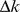 corresponding to 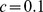 is the average value of 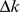 of the nodes with clustering coefficient in 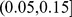. The error bars stand for standard errors.

In the first network (from 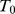 to 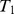), we denote 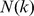 the set of nodes with degree 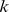. Without specific statement, a node’s degree in a directed network stands for its out-degree. Note that, we here only consider the nodes with degrees larger than 1. Denote 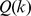 the set of node pairs 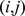 such that 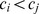 and 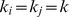, clearly, 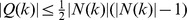. For each pair 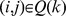, there are three cases according to 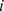
’s and 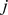
’s degrees (denoted by 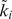 and 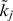) in the second network (from 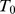 to 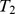): (i) 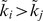 indicating that the node with lower clustering coefficient attracts more connections during the period 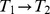; (ii) 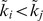 indicating that the node with higher clustering coefficient attracts more connections during the period 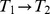; (iii) 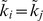 indicating that these two nodes have the same ability to attract new connections. Accordingly, we define a score 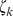 to see whether nodes with lower clustering coefficients have higher ability to attract more connections. It mathematically reads
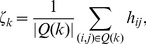
(2)where 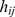 is the score depending on the aforementioned cases, as



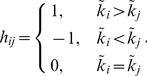
(3)Obviously, 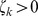 indicates that nodes having lower clustering coefficients are more likely to attract new connections than those (with the same degree) having higher clustering coefficients, while 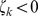 is the opposite situation. The correlation between 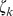 and degree 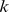 is shown in figure 1 where the area of a circle is proportional to the number of nodes with the corresponding degree. As shown in figure 1, in Cond-mat, 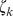 is larger than zero for 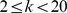 which covers 95% of all nodes with degree larger than 1 and in SM, 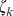 is larger than zero for 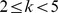 which covers 60.3% of all nodes with degree larger than 1. In addition, for small 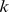, the statistics are more reliable since the number of samples is large while 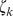 displays large fluctuations for large 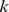 where the statistics are less reliable due to the limited statistical samples. The majority of node pairs with positive 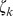 indicates that a node with smaller clustering coefficient statistically has higher ability to attract new connections. In figure 2, we show the increment of degree, 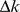, from 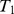 to 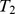. These nodes are of the same degree (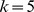) but different clustering coefficients at time 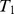. Generally speaking, 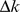 decreases with the increasing of clustering. In a word, the above empirical results (see figures 1 and 2) demonstrate that a node with smaller clustering coefficient is likely to attract more connections in the future.

### 1.2 Cluster Rank Algorithm

**Figure 3:**
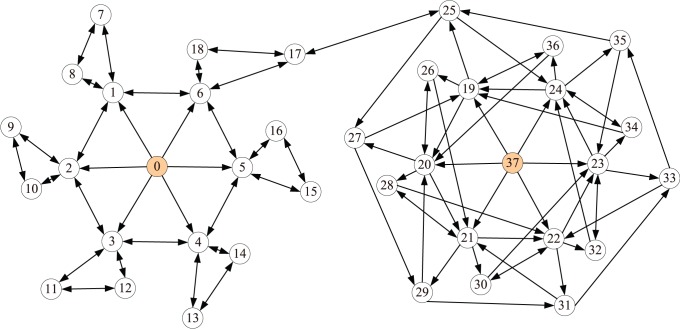
An example network with 38 nodes and 110 directed edges. Although nodes 0 and 37 have the same out-degree, node 37 is of higher influence (subject to spreading dynamics) than node 0. The clustering coefficients of these two nodes are 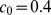 and 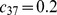.

Based on the empirical observation, we here propose a local ranking index, named ClusterRank, to quantify the influence of a node by taking into account not only its direct influence (measured by the number of its followers) and influences of its neighbors, but also its clustering coefficient. Mathematically, the ClusterRank score 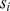 of node 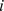 is defined as:
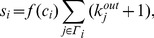
(4)where the term 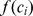 accounts for the effect of 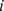
’s local clustering and the term ‘+1’ results from the contribution of 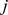 itself. Usually, the local clustering plays a negative role in spreading [28], [29], [40] since if 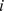
’s followers closely interact with each other rather than with other nodes, the spreading initiated from node 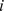 is more likely to be confined in a local region. On the contrary, if 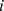
’s neighbors are mostly connected with nodes other than 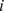
’s neighbors, the information will quickly spread to a large scope. For example, in figure 3, although node 0 has the same out-degree with node 37, node 37, with lower clustering, is of higher influence than node 0, since most of node 37’s neighbors point to nodes other than themselves and thus can send the information to wide audiences. We here adopt a simple exponential function, namely 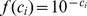, a decreasing function of 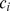. Actually, we can apply a more complicated form by introducing a new parameter, such as 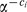 or 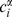. However, it adds little value to rank nodes but make the analysis more complicated. Indeed, the perspective and results of this paper are not limited by a very specific function of 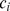.

For comparison, we briefly introduce two benchmark ranking algorithms on directed networks, PageRank [18] and LeaderRank [7]. PageRank is depicted as a random walk on hyperlinked networks. Each web page (i.e., a node) is assigned a score according to its relative importance. A parameter 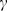 is introduced as the probability for which a web page surfers to jump to a random web page, and for probability 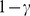 a web page surfers to continue browsing through hyperlinks. Therefore, in our case the score 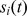 for node 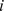 at time step 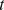 is given by:
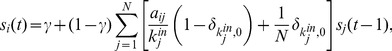
(5)where 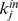 is the in-degree of node 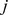 (i.e., the number of leaders of node 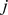), 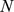 is the number of nodes of the network, 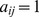 if there exists a link from 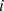 to 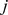 (indicating the information flow is from 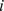 to 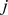), otherwise 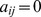, and 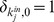 if 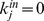, otherwise 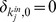. Initially, 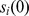 is set to be 1 for each node 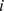, and the parameter 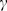 is always fixed as 0.15 in the experiments.

LeaderRank is also a random-walk-based ranking algorithm [7]. On the basis of PageRank, LeaderRank introduces a ground node 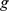, which has two directed links 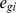 and 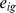 to every node 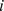 in the original network, so that the network will become strongly connected. The score 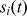 of node 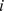 at time 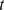 is given by (according to a purely random walk process):
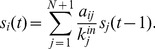
(6)


Initially, 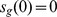 for the ground node 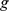, and 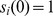 for every other node 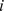. At the steady state, the score of the ground node is equally distributed to all other nodes to conserve scores on the nodes of interest. Therefore, the final score of node 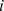, called its leadership score, is defined as
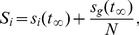
(7)where 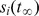 is the score of node 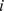 in the steady state according to Eq. (6). Notice that, although LeaderRank is similar to PageRank, it is able to dig out more influential nodes and is more stable to noise and more robust to attacks than PageRank [7]. More significantly, LeaderRank is a parameter-free ranking method. Comparing with PageRank, LeaderRank just introduces a small modification yet leads to considerable improvements.

### 1.3 Data Description

To evaluate influences of different groups of top-ranked nodes respectively obtained by out-degree centrality, PageRank, LeaderRank, 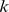-core decomposition and ClusterRank, experiments are carried out on two real directed social networks and two undirected networks. (i) Delicious: a directed social network extracted from the web site delicious.com, where the primary function of users is to collect useful bookmarks with tags. Users can select other users to be their “opinion leaders” of web browsing, in the sense that the bookmarks of the leaders are often useful and relevant. The subscriptions to leaders’ bookmarks can be made automatically. Of course users who select their leaders can in turn be the leaders of others. In that way, the users form a large-scale directed social network with information flows from leaders to followers. (ii) SM: a directed short-message communication network of a mobile company in 31 days from Dec. 8th, 2010 to Jan. 7th, 2011. In this network, each node corresponds to a mobile phone number, and a link from 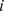 to 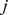 means that 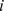 has sent at least one short message to 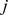 during these 31 days. We are interested in this data set because the information such as rumor may spread out in this communication network via message forwarding and influential spreaders play an important role in the spreading process. (iii) Cond-mat: a collaboration network of scientists who have posted preprints on the condensed matter archive at www.arxiv.org between Jan. 1st, 1995 and Jun. 30th, 2003. In this network, a node represents an author, and an edge connecting two authors if they have co-authorized at least one paper. The academic perspectives and the news of academic activities may propagate in this collaboration network and some key authors play the central role in the propagation. (iv) DeliciousUN: the undirected version of Delicious network where the directed links are transformed into undirected links. Some basic statistical features of these four networks, including the number of nodes, the number of links, maximum out-degree (or maximum degree for undirected network) 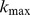, average out-degree (or average degree for undirected network) 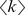 and average clustering coefficient 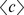, are shown in Table 1.

**Table 1:** Basic statistical features of Delicious, SM and Cond-mat networks.

Network	# nodes	# links	*k*_max_	〈*k*〉	〈*c*〉
Delicious	582377	1686131	2767	2.8953	0.1459
SM	9330493	23208675	4832	2.4874	0.0043
Cond-mat	30460	120029	202	7.8811	0.6461
DeliciousUN	582377	1340910	11187	4.6063	0.2005

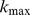
 is the maximum out-degree for directed networks or the maximum degree for undirected networks, 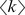 is the average out-degree for directed networks or the average degree for undirected networks, and 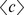 is the average clustering coefficient over all nodes.

## Results

### 2.1 Evaluation on Directed Networks

The computation times of four ranking algorithms on Delicious and SM networks are shown in Table 2. Out-degree is the fastest with runtime less than a second. Comparing with the diffusion-based methods (i.e., PageRank and LeaderRank), the time complexity of ClusterRank is much lower (a magnitude reduction). Therefore, the ClusterRank may be a promising method for very large-scale networks.

**Table 2:** The CPU time (in seconds) of out-degree centrality, PageRank, LeaderRank and ClusterRank for Delicious and SM networks in a single run.

Network	Out-degree	PageRank	LeaderRank	ClusterRank
Delicious	<1	122	646	12
SM	<1	2954	2118	191

We use C#.net language on a Core II 2.0 GHZ CPU processor with 2 GB memory.

Susceptible-Infected-Recovered (SIR) model is usually used to mimic the spreading processes of disease where infected nodes will either get immunity or die [41]. Individuals in SIR model are classified in three classes according to their states: susceptible (will not infect others but can be infected), infected (have infectivity), recovered (recovered from the illness and got immunity thus will not take part in the epidemic process, or died and thus removed from the systems). The simulation runs in discrete time steps. At each time step, every infected node randomly selects a follower and transmit the information or disease to her with probability 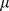 if this follower is a susceptible one. At the same time, each infected node recovers with probability 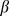, and the infected rate 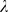 is defined as 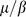. The simulation stops when there is no infected node anymore. Notice that this model is slightly different from the standard SIR model where all the followers of an infected node have the chance to be infected. The present mechanism is usually used to mimic the limited spreading capability of individuals [42], [43].

To investigate the ability of identifying influential nodes of a ranking method, we focus on top-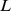 ranked nodes by out-degree centrality. Here we set 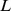 = 20 and 50 as two examples. The ranks of these 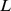 nodes by other ranking methods can be obtained via selecting them from the whole ranking lists. Then we can calculate the correlation between each pair of ranking methods by Kendall’s tau, as shown in Table 3. It can be seen that LeaderRank and PageRank are highly correlated. The correlation between ClusterRank and out-degree centrality is low in Delicious while relatively high in SM, this is because of the small clustering coefficient of SM which makes 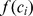 play little role in Eq. (4). For the 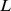 nodes with maximal out-degrees, we also investigate the correlation between the ranking scores provided by different methods and the real spreading abilities, see Table 4. The ratio between the number of infected and recovered nodes and the total number of nodes at time 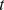, denoted by 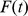, can be considered as an indicator to evaluate the influence at time 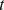. Clearly, 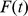 increases with 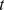, and eventually gets steady. The final coverage 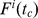 of node 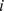 is used to represent the real spreading ability of 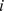 where 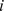 is set to be infected initially. Higher 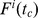 indicates higher influence of node 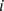. Overall speaking, the Kendall’s tau for ClusterRank is the largest.

**Table 3:** Ranking correlation measured by Kendall’s tau between different methods.

Network	CR-DR	CR-LR	CR-PR	LR-DR	LR-PR	PR-DR
Delicious Top-20	0.2211	0.6000	0.4842	0.5789	0.8632	0.5895
Delicious Top-50	0.3420	0.5711	0.4531	0.5559	0.8237	0.5722
SM Top-20	0.8895	0.9211	0.9105	0.8158	0.9895	0.8053
SM Top-50	0.6490	0.7992	0.7257	0.5510	0.9233	0.5918

Here we focus on the ranks of the top-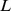 (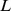 = 20 and 50) nodes with maximal out-degrees. We abbreviate ClusterRank, LeaderRank, PageRank and Out-degree centrality by CR, LR, PR and DR, respectively.

**Table 4:** Kendall’s tau between ranking scores provided by different methods and the real spreading abilities.

Network	CR	LR	PR	DR
DeliciousTop-20	0.4632	0.1263	0.0737	−0.0632
DeliciousTop-50	0.2784	0.0482	−0.0596	−0.1004
SM Top-20	0.2474	0.2368	0.2263	0.1421
SM Top-50	0.2620	0.2922	0.2253	−0.0580

Here we focus on the ranks of the top-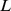 (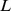 = 20 and 50) nodes with maximal out-degrees. We abbreviate ClusterRank, LeaderRank, PageRank and Out-degree centrality by CR, LR, PR and DR, respectively.

**Figure 4:**
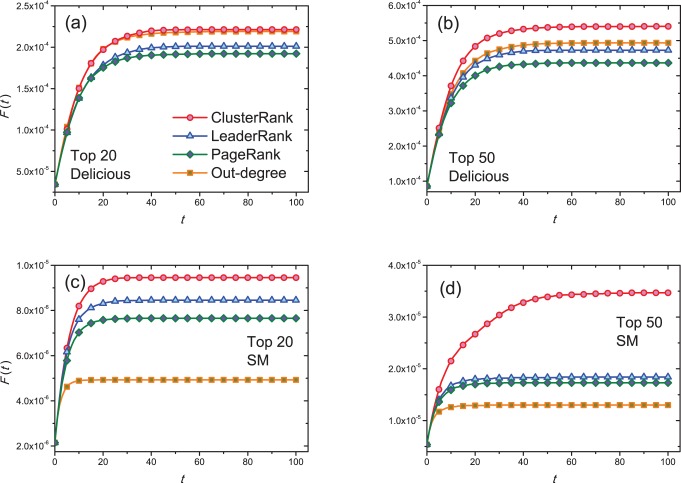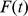 for top-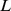 ranked nodes by out-degree centrality (squares), PageRank (diamond), LeaderRank (triangle) and ClusterRank (circles). We set 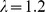 and 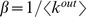. Each data point is obtained by averaging over 100 independent runs.

To investigate the influence of a group of nodes in information spreading, we initially set these nodes to be infected. We use the steady value, 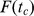, to evaluate the eventual influence of these initially infected nodes. Higher 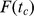 indicates higher influence. We choose the top-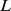 (this paper considers 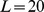 and 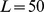) ranked nodes, which are respectively identified by out-degree centrality, PageRank, LeaderRank and ClusterRank, and set them as initially infected nodes in the experiments. Figure 4 compares 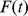 with the top-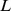 ranked nodes as the initially infected ones by out-degree, PageRank, LeaderRank and ClusterRank for Delicious and SM networks. From figure 4, one can see that the initial seeds obtained by ClusterRank result in faster and wider spreading than by other ranking methods.

**Figure 5:**
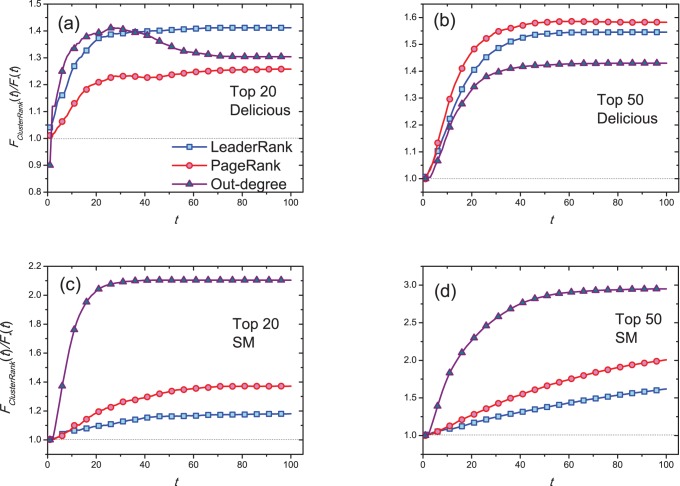
The ratio of the number of infected and recovered nodes by ClusterRank to those by out-degree centrality, PageRank and LeaderRank. Initially only non-overlapped nodes in the top-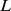 lists obtained by ClusterRank and other ranking algorithms are set to be infected. We set 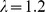 and 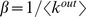. Each data point is obtained by averaging over 100 independent runs.

Since there are a considerable number of overlapped nodes in top-ranked lists of any two algorithms (see Table 5), we next compare the spreading processes resulted from non-overlapped nodes in the top-ranked lists. That is, each time when we compare the ClusterRank and another algorithm, the nodes appeared in only one list are set to be the initially infected ones. For example, for Delicious, considering the top-20 lists for out-degree centrality and ClusterRank, there are 8 non-overlapped nodes, we compare the spreading processes respectively resulted from the 8 nodes appeared only in the list by ClusterRank and the 8 nodes appeared only in the list by out-degree centrality. Figure 5 shows the ratio between the total number of infected and recovered nodes of ClusterRank and those of the other ranking algorithms, namely 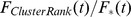, where 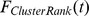 is the ratio of the total number of infected and recovered nodes to all nodes at time 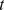 for ClusterRank, and 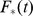 stands for the corresponding quantity of the compared algorithm (i.e., out-degree centrality, PageRank or LeaderRank). Therefore, the degree to which 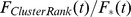 exceeds 1 indicates how much better ClusterRank performs than other methods. From figure 5, one can see that in most cases the ratio is obviously larger than 1.

**Table 5:** The number of different nodes in the top-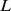 lists between ClusterRank and other three methods for Delicious and SM networks.

	Delicious		SM	
	top-20	top-50	top-20	top-50
Out-degree	8	20	19	43
PageRank	11	25	14	34
LeaderRank	7	17	17	37

**Figure 6:**
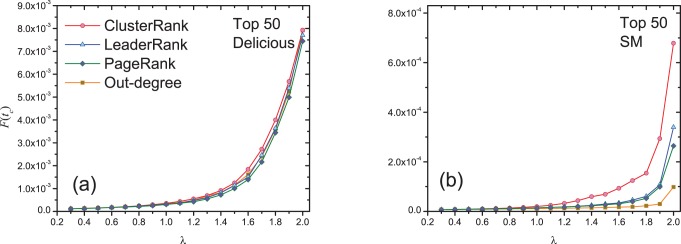
The dependence of 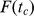 on parameter 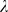. The initially infected nodes are the top-50 nodes obtained by out-degree centrality (squares), PageRank (diamonds), LeaderRank (triangles) and ClusterRank (circles). We set 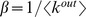. Each data point is obtained by averaging over 100 independent runs.

**Figure 7:**
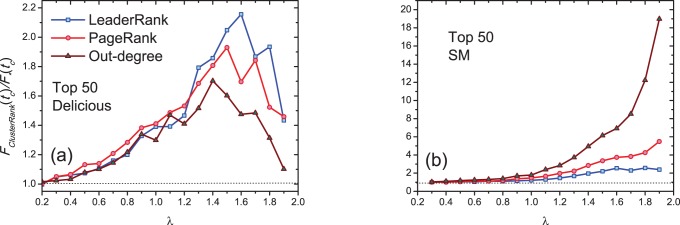
The ratio of the number of final recovered nodes by ClusterRank to those by out-degree centrality, PageRank and LeaderRank. The non-overlapped nodes in the top-50 lists are initially infected. We set 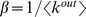. Each data point is obtained by averaging over 100 independent runs.

Figure 6 shows 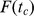 resulted from the top-50 most influential nodes at different infected rates 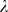. It can be seen that 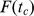 resulted from the top-50 most influential nodes by ClusterRank is larger than that by other ranking algorithms. Figure 7 shows the ratio of the number of ever infected (i.e., finally recovered) nodes resulted from top-ranked nodes by ClusterRank to those by other ranking algorithms at different infected rates 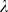. Note that, in figure 7, only non-overlapped node appeared in the top-50 lists by ClusterRank and other ranking algorithms are initially set to be infected. The ratio is up to 2 when 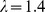 for Delicious network (see figure 7(a)) and it approaches 20 (surprisingly high) when 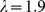 for SM network (see figure 7(b)). In fact, some nodes in the SM network are of very large out-degree but the out-degree of their followers are very small. These nodes are not as important as their out-degrees indicate, and ClusterRank could dig out really influential nodes and assign the high-degree-yet-low-influence nodes low ranks.

### 2.2 Evaluation on Undirected Networks

Above analyses show that ClusterRank is more effective than other well-known ranking methods such as out-degree centrality, PageRank and LeaderRank, in directed networks. In this subsection, we will further show the superiority of ClusterRank on undirected networks by comparing it with degree centrality and *k*-core decomposition. Here, we don’t consider PageRank and LeaderRank because they all degenerate to degree centrality in undirected networks. We use two types of initialization for SIR experiments. In the first case, we directly set the top-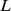 (we set 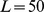 in the experiment) ranked non-overlapped nodes to be initially infected regardless of how they connect with each other. The selection method for initial seeds is similar to what we have used in figure 5. In the second case, we only consider a group of nodes with no connection between any two of them as initial seeds. Specifically, there are two steps. In the first step, for each ranking method, we select 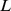 nodes who are highly ranked nodes but not connected with each other according to the following process: (i) Select the top ranked node 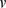 in the current network; (ii) Remove 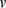 and all her neighbors from the network; (iii) Repeat step (i) and step (ii) until 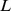 nodes have been selected. The second step is to identify the non-overlapped nodes between ClusterRank and other compared methods. For more details of how to select the initial seeds, readers could refer to Ref. [44].

**Figure 8:**
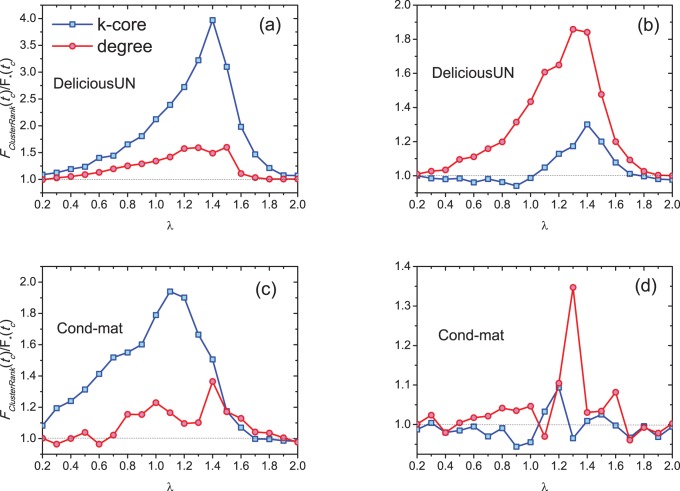
The dependance of 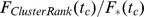 on parameter 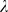 in undirected Delicious and Cond-mat networks. We set 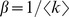. In (a) and (c), the initial infected nodes are those non-overlapped nodes in the top-50 places regardless of whether they are connected or not. In (b) and (d), the initial infected nodes are the non-overlapped nodes in top-50 places under constraint that any two of them are not connected. Each data point is obtained by averaging over 100 independent runs.

Figure 8 shows the dependence of 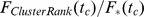 on 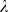 for the undirected Delicious network and Cond-mat network, where 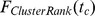 is the ratio of the total number of infected and recovered nodes to all nodes at time 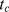 for ClusterRank, and 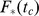 stands for the corresponding quantity of degree centrality or *k*-core decomposition. For the first case, see figures 8(a) and 8(c), the eventually infected size of ClusterRank is larger than that of degree centrality and *k*-core decomposition. In DeliciousUN, the largest value for *k*-core decomposition is 3.97 which is about 2.5 times larger than that for degree centrality. This reminds us that as a group of initial infected nodes, *k*-core decomposition may perform even worse than degree centrality [6], since the selected nodes identified by *k*-core decomposition are usually in the same core and thus densely connected with each other while the nodes selected by degree centrality or ClusterRank are usually located at different cores and thus sparsely connected. Apparently, ClusterRank is much more advanced than degree centrality. Similar results are also found in Cond-mat network, see figure 8(c). Note that, Cond-mat network is highly clustered with clustering coefficient 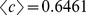, because there are many cliques each of which is constituted by a group of co-authors of a paper. Therefore the authors whose collaborators closely collaborate with each other will be highly depressed by ClusterRank due to their high clustering coefficients. The researcher with diverse collaborators who are usually belong to different communities will be more influential than those who only collaborates with people in one community. For the second case, with the consideration of the nodes that are not directly connected with each other the performance of 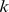-core decomposition is improved. Specifically, in DeliciousUN, ClusterRank performs much better than degree centrality especially for the middle region of 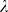 and better than that of *k*-core decomposition for 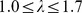. In Cond-mat network, the results of ClusterRank are still better than degree centrality and 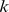-core decomposition in the middle region of 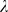, and for other region, their performances are comparable. The investigations for very small or very large infected probability 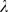 are meaningless. When 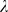 is too small (e.g., 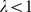), it will be hardly spread out from any group of initial nodes, and for large 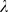, most of the nodes will get infected and thus the difference resulted from initialization will become less significant. The results shown in figure 8 demonstrate that ClusterRank also performs better than degree centrality and *k*-core decomposition in undirected networks.

## Discussion

Identifying most influential nodes in very large-scale directed networks is a key issue in network analysis, disease control, and so on. An effective and efficient ranking algorithm is proposed in this paper which emphasizes the negative effects of local clustering on spreading dynamics. Experimental results on Delicious and SM networks demonstrate that the information can spread more quickly and broadly from top-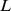 nodes obtained by our method than that by out-degree centrality, PageRank or LeaderRank. Furthermore, the method presented in this paper can be easily extended to undirected networks, for which PageRank and LeaderRank all degenerate to degree centrality. Experiments on the Cond-mat and undirected Delicious networks show that the performance of our method is also better than that of degree centrality and *k*-core decomposition for undirected networks.

How to effectively and efficiently identify influential nodes in very large-scale networks is a long-standing challenge. Lastly we list some open issues that may become the near-future focuses in this field. (1) **Algorithms from general to specific**. With different motivations and requirements, the ranking methods should be different. In our paper, we applied SIR model to evaluate the ranking performance, which actually implies that we want to find influential nodes for this specific dynamic process–the information spreading in the SIR matter. With this motivation, we find that ClusterRank is very effective. Some recent studies [30], [45] showed that in the presence of social reinforcement, the clustering may to some extent accelerate behavior propagation in online social networks. In this case, or the cases asking for critical nodes in synchronization and transportation, the ClusterRank may not be as effective as in the current case (or may be even more powerful). In real systems, users may have different preference on different topics, a topic-related ranking method will be more appropriate [46]. Furthermore, different individuals may influence other individuals through different relationships, how to make use of profiles of individuals in ranking algorithms is also interesting and challenging [8]. (2) **Algorithms on disparate types of networks**. With different network structures, suitable ranking methods might also be different. Besides the simple undirected and directed networks, ranks are required for more complicated networks including weighted networks [47], bipartite networks, multi-level networks, temporal networks [48], networks with community structure [49], and so on. Some progress has been made in this direction [50], but systematic analyses are still lacking.
